# Monosodium Urate in the Presence of RANKL Promotes Osteoclast Formation through Activation of c-Jun N-Terminal Kinase

**DOI:** 10.1155/2015/597512

**Published:** 2015-08-12

**Authors:** Jung-Yoon Choe, Ki-Yeun Park, Seong-Kyu Kim

**Affiliations:** ^1^Division of Rheumatology, Department of Internal Medicine, Catholic University of Daegu School of Medicine, Daegu 705-718, Republic of Korea; ^2^Arthritis and Autoimmunity Research Center, Catholic University of Daegu, Daegu 705-718, Republic of Korea

## Abstract

The aim of this study was to clarify the role of monosodium urate (MSU) crystals in receptor activator of nuclear factor kB ligand- (RANKL-) RANK-induced osteoclast formation. RAW 264.7 murine macrophage cells were incubated with MSU crystals or RANKL and differentiated into osteoclast-like cells as confirmed by staining for tartrate-resistant acid phosphatase (TRAP) and actin ring, pit formation assay, and TRAP activity assay. MSU crystals in the presence of RANKL augmented osteoclast differentiation, with enhanced mRNA expression of NFATc1, cathepsin K, carbonic anhydrase II, and matrix metalloproteinase-9 (MMP-9), in comparison to RAW 264.7 macrophages incubated in the presence of RANKL alone. Treatment with both MSU crystals and RANKL induced osteoclast differentiation by activating downstream molecules in the RANKL-RANK pathway including tumor necrosis factor receptor-associated factor 6 (TRAF-6), JNK, c-Jun, and NFATc1. IL-1b produced in response to treatment with both MSU and RANKL is involved in osteoclast differentiation in part through the induction of TRAF-6 downstream of the IL-1b pathway. This study revealed that MSU crystals contribute to enhanced osteoclast formation through activation of RANKL-mediated pathways and recruitment of IL-1b. These findings suggest that MSU crystals might be a pathologic causative agent of bone destruction in gout.

## 1. Introduction

Osteoclasts are considered crucial effector cells that are mainly responsible for bone resorption in bone homeostasis [1–3]. Receptor activator of nuclear factor-*κ*B ligand (RANKL) and its receptor RANK are components in a signaling pathway that is essential for osteoclast differentiation, activation, and survival [1–3]. The involvement of mature, multinucleated osteoclasts is essential in explaining bone damage in chronic autoimmune inflammatory arthritides such as rheumatoid arthritis (RA) [4] and psoriatic arthritis [5].

Similarly, enhanced osteoclastogenesis mediated by urate crystals was observed in patients with chronic erosive gouty arthritis [6, 7]. Tophi in chronic gout have been identified as a characteristic presenting feature with foreign body granuloma composed of mono- and multinucleated macrophages around the deposit of monosodium urate (MSU) crystals [8, 9]. Two recent studies identified numerous multinucleated osteoclast-like cells expressing osteoclast phenotypic markers such as cathepsin K, CD51, and tartrate-resistant acid phosphatase (TRAP) within tophi and at the interface between bone and soft tissue [6, 7]. Another study using quantitative immunohistochemistry analysis found numerous CD68+ and TRAP+ multinucleated cells (TRAP+ MNCs) within the cellular (or corona) zone surrounding MSU crystals in tophi [10]. In addition, higher levels of serum RANKL, macrophage colony-stimulating factor (M-CSF), and soluble interleukin-6 (IL-6) receptor, which is also related to bone metabolism, were found in patients with chronic erosive gouty arthritis, rather than nonerosive gout [6, 11]. These findings suggest that the interaction between osteoclasts and urate crystals plays a key role in the development of bone damage in gouty arthritis.

It has not been clearly determined for molecular mechanism of MSU crystals on osteoclast formation in the downstream of RANKL-RANK pathway. In this study, we investigated whether MSU crystals in the presence of RANKL promote differentiation of murine RAW 264.7 macrophages into osteoclasts. We also sought to identify the role of cytoplasmic protein kinases and transcription factors downstream of the RANKL-RANK signaling pathway and of IL-1*β* in osteoclast formation.

## 2. Materials and Methods

### 2.1. Cell Culture

Murine monocyte/macrophage RAW 264.7 cells were purchased from the Korean Cell Line Bank (KCLB, Seoul, Korea) and maintained in Dulbecco's modified Eagle's medium (DMEM) (Gibco BRL, Grand Island, NY, USA) supplemented with 10% fetal bovine serum (Hyclone, Logan, USA), 100 U/mL penicillin, and 100 *μ*g/mL streptomycin. The subcultures were performed when cells reached a confluence of 80~90%. MSU crystals are prepared by methods of our previous study [12].

### 2.2. Quantitative Real Time-Polymerase Chain Reaction (qRT-PCR)

Total RNA was extracted from 5 × 10^5^ cells using TRIzol reagent (Invitrogen, Carlsbad, CA, USA) according to the manufacturer's instructions. Complementary DNAs (cDNA) were synthesized from 1 *µ*g of total RNA using ReverTra Ace-*α*-kit (Toyobo, Osaka, Japan). Briefly, RNA was mixed with ReverTra Ace, 5x RT buffer (contains 25 mM Mg_2_^+^), RNase inhibitor (10 U/*μ*L), dNTPs mixture (10 mM), Oligo(dT)20 (10 pmol/*μ*L), and RNase-free water containing final volume of 20 *µ*L mixture. Reverse transcription was incubated at 42°C for 20 min, followed by heating at 99°C for 5 min, and stored at 4°C.

Real-time PCR was performed using the MiniOpticon real-time PCR system (Bio-Rad, Hercules, California, USA) with SYBR Green PCR Master Mix (Toyobo, Osaka, Japan) according to the manufacturers' instructions. The reaction was performed in a total volume of 20 *µ*L containing 10 *µ*L of SYBR Green real-time PCR Master Mix, 10 pmol/L of each primer, 2 *µ*L of cDNA, and 6.4 *µ*L of distilled water. After that PCR protocols were followed: 50°C for 2 min 95°C for 10 min and 40 cycles of 15 s, 95°C/1 min, 72°C/45 s; and 60°C to 95°C per cycle for melting curve analysis. Primers for the following were synthesized by Bionics (Seoul, Korea): RANKL: forward 5′-TAC TTT CGA GCG CAG ATG GAT-3′; reverse 5′-ACC TGC GTT TTC ATG GAG TCT-3′; RANK: forward 5′-CCA GGA CAG GGC TGA TGA GAA-3′; reverse 5′-TGG CTG ACA TAC ACC ACG ATG A-3′; cathepsin K: forward 5′-CAG CAG AAC GGA GGC ATT GA-3′; reverse 5′-CCT TTG CCG TGG CGT TAT AC-3′; carbonic anhydrase II: forward 5′-CAT TAC TGT CAG CAG CGA GCA-3′; reverse 5′-GAC GCC AGT TGT CCA CCA TC-3′; matrix metalloproteinase-9 (MMP-9): forward 5′-GCC CTG GAA CTC ACA CGA CA-3′; reverse 5′-TTG GAA ACT CAC ACG CCA GAA-3, and GAPDH: forward 5′-AAG GCT GTG GGC AAG GTC ATC-3′; reverse 5′-CAG GCG GCA CGT CAG ATC C-3′.

### 2.3. Western Blot Analyses

Cell (4 × 10^6^) pellets were lysed in lysis buffer (1 M Tris–HCl pH 8.0, 5 M NaCl, 10% Nonidet P40, and protease inhibitor cocktail 1 tablet (Roche Diagnostics, Mannheim, Germany)) and incubated on ice for 10 min and centrifuged at 12,000 rpm for 10 min at 4°C. The pellet was discarded and total protein concentration in the supernatant was determined using Bio-Rad protein assay kit (Bio-Rad, Hercules, CA, USA). Proteins (40–60 *μ*g) were separated by 10% SDS-PAGE gel electrophoresis and then transferred to a nitrocellulose membranes (Bio-Rad, Hercules, CA, USA) and probed with appropriate antibodies. Antibodies to tumor necrosis factor receptor-associated factor 6 (TRAF-6), JNK, phospho-c-Jun, nuclear factor of activated T-cells cytoplasmic 1 (NFATc1), and *β*-actin from Santa Cruz Biotechnology (Santa Cruz, CA, USA), phospho-p38 and p38 from Cell Signaling Technology (Beverly, MA, USA), and phospho-ERK, phospho-JNK, and IL-1*β* from Abcam (Cambridge, UK) were purchased. Primary antibodies were incubated overnight at 4°C and horseradish peroxidase-conjugated secondary antibodies were incubated for 1 h at room temperature. Cells (4 × 10^6^) pretreated with specific mitogen-activated protein kinase (MAPK) inhibitors such as U0126 for ERK, SP600125 for JNK, and SB203580 for p38 (Sigma, St. Louis, MO, USA) for 1 h were incubated with MSU crystals (0.1 mg/mL) alone, RNAKL (100 ng/mL) alone, or both for 72 h. Proteins were detected with the SuperSignal West Pico chemiluminescent kit (Thermo Scientific, Rockford, IL, USA). Densitometry were analyzed and quantified with Quantity One software (Bio-Rad, Hercules, CA, USA).

### 2.4. Transfection of Small Interfering RNA (siRNA)

Cells were seeded in 1 × 10^5^ cells per 24-well plates and transfected with 50 ng/mL of siRNA for each target gene including mouse IL-1*β* and mouse TRAF-6 (Invitrogen, Carlsbad, CA, USA) using Lipofectamine RNAiMAX Reagent (Invitrogen, Carlsbad, CA, USA) in Opti-MEM media (Gibco BRL). Negative siRNA control used was nontargeting negative control siRNA (Med GC; Invitrogen, Carlsbad, CA, USA). Also, negative siRNA control was used to refer to “mock transfected cells” in manuscripts. The mixtures were combined for 15 min at room temperature. After 72 h of incubation, transfected cells were harvested.

### 2.5. TRAP Staining Assay

Cells were incubated in 24-well plates at 1 × 10^4^ cells and treated with 100 ng/mL of RANKL in the absence or presence of MSU crystals. Osteoclast differentiation was performed using TRAP staining kit (Takara Bio Inc., Otsu, Shiga, Japan). Briefly, cells were fixed with fixative solution for 5 min and washed in distilled water. Cells were stained with acid phosphatase containing tartrate-resistant enzyme, 0.1 volume of sodium tartrate, and the mixture was incubated at 37°C for 40 min. Then cells were washed with distilled water. TRAP+ MNCs with at least 3 or more nuclei were defined as osteoclasts. TRAP+ MNCs were observed using a light microscope.

### 2.6. Actin Ring Staining

Cell were seeded in 24-well plates at 1 × 10^4^ cells and incubated with 100 ng/mL of RANKL in the absence or presence of MSU crystals. Actin ring was formed using FITC-phalloidin (Santa Cruz, CA, USA) staining. Cells were fixed with 4% paraformaldehyde in PBS for 10 min. And cells were incubated with FITC-phalloidin in PBS for 40 min at room temperature. After cells were washed with PBS, the actin ring was visualized fluorescently using a microscopy (TE2000-U, Nikon Instruments Inc., NY, USA).

### 2.7. TRAP Solution Assay for TRAP Activity

For the TRAP solution assay, cells were cultured in 96-well plates and incubated with MSU crystals and RANKL for various times. Then, cells were lysed with 100 *μ*L of lysis buffer (1% Triton X-100, 120 mg sodium acetate in 120 mM sodium acetate buffer) for 5 min. The supernatants were transferred to a new 96-well plate adding* p*-nitrophenyl phosphate. After the incubation, solution was added to 1 M NaOH and the absorbance was measured using a microplate reader at 405 nm.

### 2.8. Statistical Analysis

Data were expressed as mean ± standard error of mean in 3 independent experiments. Experimental results were analyzed using the Mann-Whitney test and analysis of variance (ANOVA) test, if appropriate. Data were analyzed using the SPSS statistical program version 13.0 for Windows (SPSS Inc., Chicago, IL, USA). *p* values less than 0.05 were considered statistically significant.

## 3. Results

### 3.1. Costimulation with MSU Crystals and RANKL-Induced RAW 264.7 Macrophages to Differentiate into Osteoclasts

**Figure 1:**
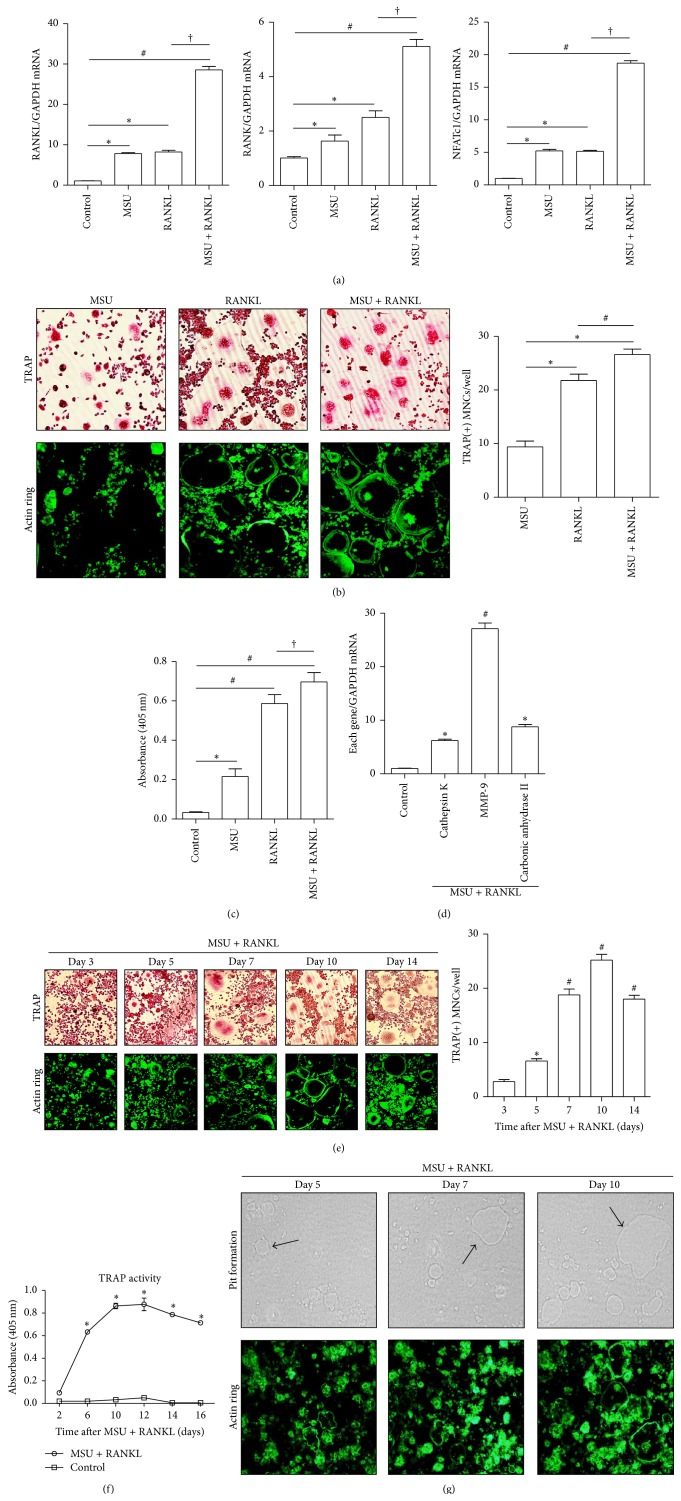
Osteoclast formation induced by stimulation with MSU crystals in the presence of RANKL. (a) RAW 264.7 cells alone (controls) were incubated with MSU crystals alone (0.1 mg/mL), RANKL alone (100 ng/mL), or both for 24 h. ^*∗*^ *p* < 0.05, ^#^ *p* < 0.01, and ^†^ *p* < 0.01. (b) TRAP staining and actin ring staining were performed in cells incubated with MSU crystals alone (0.1 mg/mL), RANKL alone (100 ng/mL), or both for 10 days. ^*∗*^ *p* < 0.01 and ^#^ *p* < 0.05. (c) Production of TRAP protein was measured using the TRAP activity assay. RAW 264.7 cells alone (controls) were incubated with MSU crystals alone (0.1 mg/mL), RANKL alone (100 ng/mL), or both for 24 h. ^*∗*^ *p* < 0.05, ^#^ *p* < 0.01, and ^†^ *p* > 0.05. (d) The mRNA expression of osteoclast-specific genes was measured. RAW 264.7 cells were treated with both MSU crystals (0.1 mg/mL) and RANKL (100 ng/mL) for 24 h. ^*∗*^ *p* < 0.05 and ^#^ *p* < 0.01. (e) TRAP and actin ring staining were performed after stimulation of RAW 264.7 cells with both MSU crystals (0.1 mg/mL) and RANKL (100 ng/mL). ^*∗*^ *p* < 0.05 and ^#^ *p* < 0.01. (f) TRAP activity was measured and compared between RAW 264.7 cells incubated with both MSU crystals (0.1 mg/mL) and RANKL (100 ng/mL) and control cells. ^*∗*^ *p* < 0.01 by ANOVA. (g) Functional assessment for activated osteoclasts was performed using the pit formation assay. The assay was performed at days 5, 7, and 10 after stimulation with both MSU crystals (0.1 mg/mL) and RANKL (100 ng/mL). Arrows indicate pits formed by activated osteoclasts. The experimental data were determined by at least three independent tests.

Initially we assessed whether MSU crystals and/or RANKL activate genes related to RANKL/RANK-mediated osteoclast formation. A quantitative PCR assay demonstrated that MSU crystals alone or RANKL alone induced mRNA expression of RANKL, RANK, or NFATc1 genes in comparison to nontreated RAW 264.7 cells (*p* < 0.05 for each gene) (Figure 1(a)). RAW 264.7 cells incubated with both MSU crystals and RANKL had significantly increased RANKL, RANK, and NFATc1 mRNA expression compared to controls (*p* < 0.01 for each gene). There was also marked differences in the expression of these genes between cells costimulated with both MSU crystals and RANKL and RANKL alone (*p* < 0.01 for each gene).

As shown in Figure 1(b), osteoclast-like cells were rarely detected among RAW 264.7 cells treated with only MSU crystals, which is consistent with the findings of a previous in vitro study [6]. However, treatment with RANKL alone and with both MSU crystals and RANKL sufficiently induced the formation of more TRAP+ MNCs (*p* < 0.01) and more prominent actin ring+ cells than treatment with MSU crystals alone. Significantly enhanced formation of TRAP+ MNCs and actin ring+ cells was observed after treatment with both MSU crystals and RANKL compared to RANKL alone (*p* < 0.05). In addition, the TRAP activity assay illustrated that treatment with either MSU crystals or RANKL alone or both together resulted in the production of much more TRAP protein than controls (*p* < 0.05, *p* < 0.01, and *p* < 0.01, resp.) (Figure 1(c)). However, TRAP activity was found to be similar between cells treated with both MSU crystals and RANKL and those treated with RANKL alone (*p* > 0.05). In terms of the expression of osteoclast-specific genes involved in osteoclast activation, the mRNA expression of cathepsin K, MMP-9, and carbonic anhydrase II was significantly enhanced in comparison to expression in control macrophages (*p* < 0.05, *p* < 0.01, and *p* < 0.05, resp.) (Figure 1(d)).

Both TRAP+ and actin ring+ cells differentiated from RAW 264.7 macrophages cultured with MSU crystals in the presence of RANKL were detected from day 5 of incubation (Figure 1(e)). Thereafter the number of these cells gradually increased in a time-dependent manner for up to 10 days of incubation.  Figure 1(f) illustrates that TRAP activity in cells cultured with both MSU crystals and RANKL also increased in a time-dependent manner after incubation for 12 days, and thereafter activity gradually decreased up to day 16 (*p* < 0.01).

A pit formation assay was performed to assess functional bone resorption by osteoclasts differentiated from RAW 264.7 cells cultured with both MSU crystals and RANKL (Figure 1(g)). The number and size of pits formed by bone resorption gradually increased from 5 days to 10 days. In addition, larger actin ring+ cells were frequently noted among RAW 264.7 macrophages stimulated with both MSU crystals and RANKL over time.

### 3.2. MSU Crystals in the Presence of RANKL-Induced Osteoclast Differentiation through Activation of TRAF-6 and JNK

**Figure 2:**
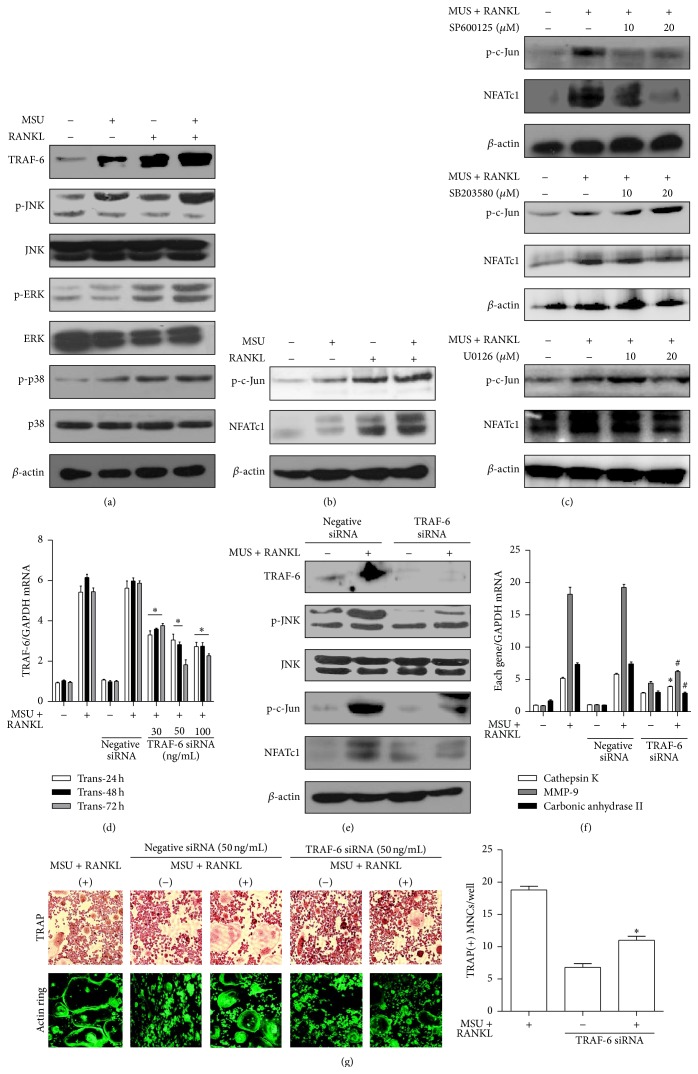
Induction of osteoclast differentiation through activation of TRAF-6 and JNK. (a) The protein expression of cytoplasmic molecules of the RANKL-RANK signaling pathway was assessed in RAW 264.7 cells alone (controls) and experimental cells incubated with MSU crystals alone (0.1 mg/mL), RANKL alone (100 ng/mL), or both for 1 h. (b) Phospho-c-Jun and NFATc1 production was measured in RAW 264.7 cells alone (controls) and experimental cells incubated with MSU crystals alone (0.1 mg/mL), RANKL alone (100 ng/mL), or both for 24 h. (c) The levels of phospho-c-Jun and NFATc1 protein were measured using a JNK inhibitor (SP600125), p38 inhibitor (SB203580), and ERK inhibitor (U0126) at dosages of 10 and 20 *μ*M. (d) TRAF-6 mRNA expression was assessed in RAW 264.7 cells alone (controls) and cells transfected with negative siRNA and TRAF-6 siRNA at different dosages (0, 30, 50, and 100 ng/mL) and incubation times (24, 48, and 72 h) in the presence and absence of both MSU crystals (0.1 mg/mL) and RANKL (100 ng/mL). ^*∗*^ *p* < 0.05 and ^#^ *p* < 0.05 compared to cells transfected with 0 ng/mL of TRAF-6 siRNA. (e) TRAF-6, phospho-JNK, phospho-c-Jun, and NFATc1 protein levels were measured from RAW 264.7 cells transfected with negative siRNA and TRAF-6 siRNA (50 ng/mL) for 72 h. (f) The mRNA expression of osteoclast-specific genes was measured in controls and negative and TRAF-6 siRNA (50 ng/mL) RAW 264.7 cells. ^*∗*^ *p* < 0.05 and ^#^ *p* < 0.01 compared to cells treated with both MSU crystals and RANKL. (g) TRAP and actin ring staining assays for osteoclast-like cells were performed in control RAW 264.7 cells and TRAF-6 siRNA transfected cells under stimulation with both MSU crystals (0.1 mg/mL) and RANKL (100 ng/mL). ^*∗*^ *p* < 0.05 compared to control cells treated with both MSU crystals and RANKL. The experimental data were determined by at least three independent tests.

We examined whether MSU crystals and/or RANKL induced expression of the adaptor molecule TRAF-6 and MAPKs including JNK, ERK, and p38. As shown in Figure 2(a), incubation with RANKL alone and both RANKL and MSU crystals induced more prominent expression of TRAF-6 and phosphorylation of the three individual MAPK proteins compared to nontreated cells and those incubated with MSU crystals alone.

Activation of NFATc1 and c-Jun in the RANKL/RANK pathway plays a crucial role in osteoclast differentiation and activation. This study assessed protein levels of these molecules after treatment with MSU crystals and/or RANKL (Figure 2(b)). MSU crystals alone rarely induced phospho-c-Jun and NFATc1 protein expression. However, treatment with RANKL alone and both RANKL and MSU crystals enhanced phospho-c-Jun and NFATc1 production at the translational level. In addition, JNK inhibitor (SP600125) at dosages of 10 and 20 *μ*M markedly attenuated phospho-c-Jun and NFATc1 protein levels in RAW 264.7 macrophages stimulated with both MSU crystals and RANKL (Figure 2(c)). However, p38 inhibitor (SB203580) and ERK inhibitor (U0126) did not inhibit these target molecules.

Enhanced TRAF-6 mRNA expression in RAW 264.7 cells treated with RANKL and MSU crystals was markedly suppressed in cells transfected with siRNA for TRAF-6 (TRAF-6 siRNA) at three different dosages and times (Figure 2(d)). After treatment with both MSU crystals and RANKL, RAW 264.7 macrophages transfected with TRAF-6 siRNA exhibited significantly attenuated phosphorylation of JNK and c-Jun and NFATc1 protein expression compared to control macrophages (Figure 2(e)). In terms of osteoclast-specific genes, mRNA expression of cathepsin K, MMP-9, and carbonic anhydrase II was significantly suppressed in RAW 264.7 macrophages transfected with TRAF-6 siRNA (Figure 2(f)). In the TRAP staining assay, the formation of TRAP+ MNCs and actin ring+ cells after transfection with TRAF-6 siRNA was suppressed in cells treated with MSU crystals and RANKL in comparison to control cells (Figure 2(g)). The number of TRAP+ MNCs and actin ring+ cells was significantly reduced after TRAF-6 siRNA transfection compared to controls (*p* < 0.05).

### 3.3. IL-1*β* Is Required for Osteoclast Activation Mediated by MSU Crystals in the Presence of RANKL

**Figure 3:**
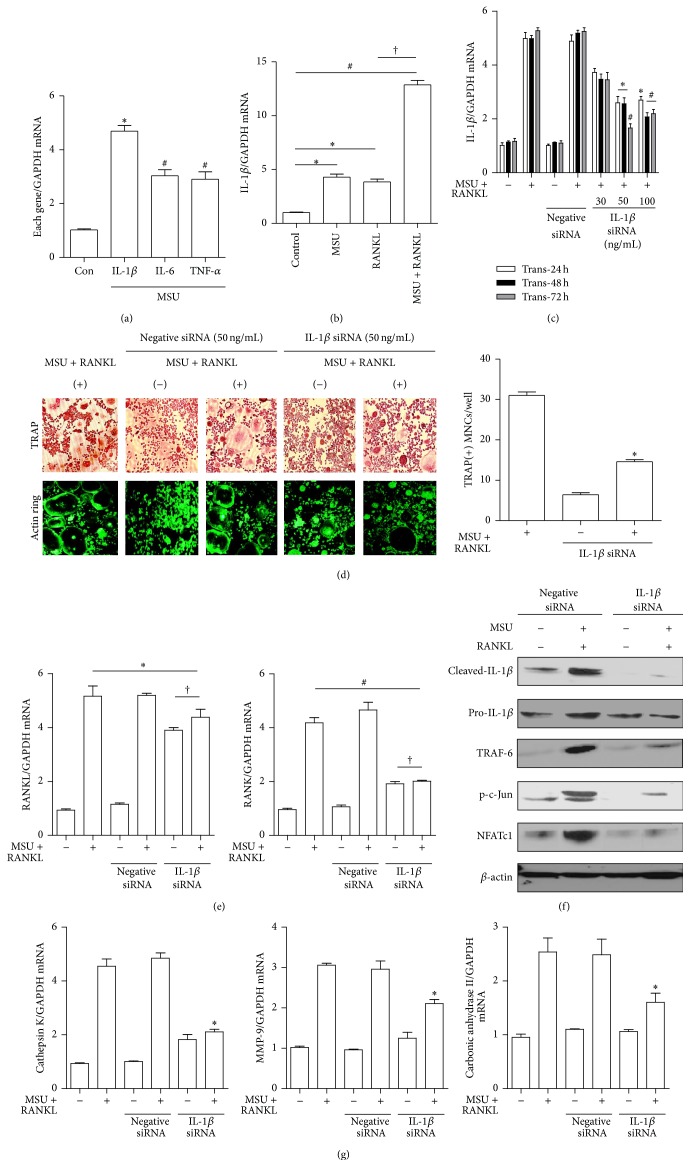
The role of IL-1*β* in osteoclast activation stimulated by MSU crystals. (a) Gene expression of proinflammatory cytokines was measured after stimulation with 0.1 ng/mL of MSU crystals for 24 h. ^*∗*^ *p* < 0.01 and ^#^ *p* < 0.05 compared to controls. (b) IL-1*β* gene expression in RAW 264.7 cells alone (controls) and cells incubated with MSU crystals alone (0.1 mg/mL), RANKL alone (100 ng/mL), or both for 24 h. ^*∗*^ *p* < 0.05, ^#^ *p* < 0.01, and ^†^ *p* < 0.01. (c) IL-1*β* mRNA expression was assessed in RAW 264.7 cells alone (controls) and cells transfected with negative and IL-1*β* siRNA at different dosages (0, 30, 50, and 100 ng/mL) and incubation times (24, 48, and 72 h) in the presence or absence of both MSU crystals (0.1 mg/mL) and RANKL (100 ng/mL). ^*∗*^ *p* < 0.05 and ^#^ *p* < 0.01 compared to cells transfected with 0 ng/mL of IL-1*β* siRNA. (d) TRAP and actin ring staining were performed in control RAW 264.7 cells and IL-1*β* siRNA (50 ng/mL) transfected cells under stimulation with both MSU crystals (0.1 mg/mL) and RANKL (100 ng/mL) for 10 days. ^*∗*^ *p* < 0.05 compared to control cells treated with both MSU crystals and RANKL. (e) RANKL and RANK gene expression were measured in controls and negative and IL-1*β* siRNA transfected cells under stimulation with both MSU crystals (0.1 mg/mL) and RANKL (100 ng/mL) for 24 h. ^*∗*^ *p* > 0.05, ^#^ *p* < 0.01, and ^†^ *p* > 0.05. (f) Cleaved IL-1*β*, TRAF-6, phospho-JNK, phospho-c-Jun, and NFATc1 protein levels were measured in RAW 264.7 cells transfected with negative siRNA and IL-1*β* siRNA (50 ng/mL) for 72 h. (g) The mRNA expression of osteoclast-specific genes was measured in controls and negative and IL-1*β* siRNA (50 ng/mL) RAW 264.7 cells. ^*∗*^ *p* < 0.05 compared to cells treated with both MSU crystals and RANKL.

We measured mRNA expression of proinflammatory cytokines including IL-1*β*, TNF-*α*, and IL-6 in response to MSU crystals and found a significant increase in these cytokines (Figure 3(a)). Incubation with MSU crystals alone, RANKL alone, or both prominently induced IL-1*β* mRNA expression, which was statistically significant compared to controls (*p* < 0.05, *p* < 0.05, and *p* < 0.01, resp.) (Figure 3(b)). In addition, costimulation with both MSU crystals and RANKL significantly induced mRNA expression of IL-1*β* compared to stimulation with RANKL alone (*p* < 0.01) (Figure 3(b)). RAW 264.7 cells transfected with siRNA for IL-1*β* (IL-1*β* siRNA) showed markedly lower IL-1*β* mRNA expression at three different dosages and times (Figure 3(c)).

In the TRAP staining assay for osteoclast formation, transfection with 50 ng/mL of IL-1*β* siRNA after stimulation with both MSU crystals and RANKL resulted in fewer TRAP+ MNCs than the control (*p* < 0.05) (Figure 3(d)). Actin ring formation in IL-1*β* siRNA transfected cells was also markedly suppressed compared to control cells.

The enhanced mRNA expression of RANKL in controls stimulated with both MSU crystals and RNAKL was not different from that in IL-1*β* siRNA RAW 264.7 cells (Figure 3(e)). In contrast, RANK expression was significantly reduced in IL-1*β* knockdown RAW 264.7 cells (*p* < 0.01). However, there were no differences in RANKL and RANK mRNA expression between IL-1*β* siRNA RAW 264.7 cells incubated in the presence and absence of MSU crystals and RANKL (*p* > 0.05).

The significant reduction of cleaved IL-1*β* protein expression was confirmed in IL-1*β* siRNA transfected RAW 264.7 cells (Figure 3(f)). Enhanced expression of TRAF-6, c-Jun, and NFATc1 induced by both MSU crystals and RANKL was markedly reduced in RAW 264.7 cells transfected with IL-1*β* siRNA compared with that in cells transfected with negative siRNA. Assessment of expression for osteoclast-specific markers revealed that IL-1*β* siRNA transfected macrophages treated with both MSU crystals and RANKL significantly inhibited mRNA expression of cathepsin K, MMP-9, and carbonic anhydrase II in comparison to cells transfected with controls (Figure 3(g)).

## 4. Discussion

Mature osteoclasts are functionally bone resorbing cells mediated by secreting proteolytic enzymes, including cathepsin K and MMPs. Several hormones and factors that stimulate bone resorption in vivo increased mRNA expression of RANKL including parathyroid hormone, 1*α*25-(OH)_2_ vitamin D3, IL-1, PGE2, and calcineurin inhibitors, whereas TGF-*β* is a downregulator [13]. Higher levels of circulating RANKL were identified in severe erosive tophaceous gout patients in comparison to patients without tophi [6]. Abundant RANKL+ and RANK+ stained cells were found around tophi or in osteolytic lesions in gouty tophaceous tissues [7]. In this study, we initially assessed whether MSU crystals could regulate expression of RANKL and its receptor RANK in RAW 264.7 cells. We found that treatment of experimental cells with MSU crystals increased mRNA expression of RANKL and RANK. This finding is consistent with previous data that showed that RANKL gene expression in PBMCs sampled from healthy subjects was markedly induced by MSU crystals [7]. In contrast, Dalbeth et al. reported that MSU crystals did not significantly induce RANKL gene expression and generation of proinflammatory cytokines such as TNF-*α*, IL-1*β*, and IL-6 in a murine bone marrow stromal cell line ST2 [6]. However, these inflammatory cytokines were prominently increased in RAW 264.7 cells in our study. The contrast in data might be due to the use of different cell lines.

It has generally been believed that concomitant treatment with RANKL and M-CSF is essential components for differentiation of monocyte/macrophage precursor cells into mature osteoclasts [1, 2]. However, the murine monocytic RAW 264.7 cells sufficiently induce osteoclast formation by RANKL without M-CSF stimulation [14, 15]. This study assessed osteoclast formation using morphologic assays by TRAP and actin ring staining methods and TRAP activity assay. Although MSU crystals showed small increase of TRAP activity, we identified inability of MSU crystals alone for formation of TRAP+ and actin ring+ osteoclast-like cells. It is more compatible to the result of previous study, showing MSU crystals not stimulating osteoclast formation [6]. As in previous studies [14, 15], we found that RANKL alone induced the differentiation of RAW 264.7 cells into osteoclast-like cells. Concomitant stimulation with both MSU crystals and RANKL markedly enhanced the formation of osteoclast-like cells in comparison to stimulation with RANKL alone as shown in Figures 1(a) and 1(b). It suggests that MSU crystals play a key role in RANKL-induced osteoclast formation as a potent inducer of RANKL and MSU crystal formation in bone damage associated with chronic gouty arthritis.

Diverse cytoplasmic factors including NF-*κ*B, MAPKs, and Src activate RANKL/RANK-mediated signaling cascades. MAPKs are involved in osteoclastogenesis through activation of the transcription factor AP-1 complex, consisting of Fos, Jun, and ATF [16]. Data indicating that mutagenesis of c-Fos results in osteopetrosis due to impaired osteoclastogenesis suggests that MAPKs have a crucial role in osteoclast formation by regulating AP-1 activity [17]. Activation of ERK-1 by MEK1 and p38 by MKK6 has been well established to be involved in the regulation of osteoclastogenesis [3]. An in vitro study demonstrated that osteoclastogenesis is partially impaired in JNK deficient cells without apparent bone abnormalities [18]. The present study showed that RANKL and MSU crystals induced activation of ERK, JNK, and p38 MAPKs. However, we found that only a specific inhibitor of JNK downregulated phospho-c-Jun and NFATc1 in RAW 264.7 cells. In contrast, specific inhibitors of p38 and ERK induced activation of phospho-c-Jun and NFATc1 in this study. Hotokezaka et al. reported that ERK inhibitors U0126 and PD98059 stimulated RANKL-induced osteoclast differentiation, suggesting that the ERK pathway is a negative regulator of osteoclastogenesis [19]. Our data implies that osteoclastogenesis induced by RANKL and MSU crystals might be mediated by activation of the JNK pathway, but not the ERK and p38 pathways.

TRAF-6, which binds to the cytoplasmic domain of RANK, is an adaptor protein that regulates cell apoptosis and proliferation through the JNK and NF-*κ*B pathways [20]. Evidence supporting the fact that TRAF-6 is a key protein in osteoclastogenesis was obtained from TRAF-6 knockout mice that developed osteopetrosis [21]. TRAF-6 is also believed to be an essential molecule for RANK-induced NK-*κ*B and JNK activation in osteoclast formation. The number of TRAP+ MNCs was significantly decreased in RAW 264.7 cells transfected with TRAF-6 siRNA after stimulation with MSU crystals and RANKL in this study, supporting the important role of TRAF-6 in osteoclastogenesis.

MSU crystals act as a danger signal and trigger innate immune responses through Toll-like receptor-2 (TLR2) and TLR4. Macrophages signaled through TLR2 and TLR4 showed significantly decreased expression of proinflammatory cytokines including IL-1*β* and TNF-*α* [22]. Intracellular engagement of TLR-mediated MSU crystals induces the release of pro-IL-*β* via NF-*κ*B activation and promotes the NALP3 inflammasome to drive caspase-1 activation, leading to secretion of active IL-1*β* [23, 24]. A recombinant IL-1 receptor antagonist (anakinra) and IL-1 TRAP were shown to be effective in patients with acute and chronic gouty inflammation [25, 26]. It has been established that IL-1*β* is a major cytokine involved in the pathogenesis of gout and is also considered a promising therapeutic target to control the inflammatory response in gout. In addition, binding of the proinflammatory cytokine IL-1 to its receptor plays a crucial role in promotion of osteoclastogenesis through activation of TRAF-6, an adaptor protein in the RANKL-RANK signaling pathway [27, 28]. A higher level of IL-1*β* in synovial fluid mononuclear cells from patients with chronic gouty arthritis was noted than that in peripheral mononuclear cells (PBMCs) [7], which suggests that IL-1*β* might be involved in osteoclast formation. We found enhanced protein expression of TRAF-6, phospho-c-Jun, and NFATc1 in response to MSU crystals and RANKL, with significant downregulation in RAW 276.4 cells transfected with IL-1*β* siRNA. In addition, the number of TRAP+ MNCs was markedly decreased in IL-1*β* siRNA cells. Interestingly, this experiment showed that RANKL gene expression was unresponsive to MSU and RANKL costimulation in IL-1*β* siRNA cells, indicating that IL-1*β* is a key cytokine driving osteoclast formation in a RANKL-RANK-independent manner.

## 5. Conclusion

The major finding of this study is that MSU crystals in the presence of RANKL augmented osteoclast formation from RAW 264.7 cells through activation of TRAF-6 to JNK. We also found that IL-1*β*, a proinflammatory cytokine involved in the pathogenesis of gout and also a preresorptive cytokine for bone metabolism, might be a potent mediator in the activation of osteoclasts, which results in the bone erosion seen in gout.

## References

[1] Takayanagi H. (2007). Osteoimmunology: shared mechanisms and crosstalk between the immune and bone systems. *Nature Reviews Immunology*.

[2] Edwards J. R., Mundy G. R. (2011). Advances in osteoclast biology: old findings and new insights from mouse models. *Nature Reviews Rheumatology*.

[3] Asagiri M., Takayanagi H. (2007). The molecular understanding of osteoclast differentiation. *Bone*.

[4] Udagawa N., Kotake S., Kamatani N., Takahashi N., Suda T. (2002). The molecular mechanism of osteoclastogenesis in rheumatoid arthritis. *Arthritis Research*.

[5] Colucci S., Brunetti G., Cantatore F. P. (2007). Lymphocytes and synovial fluid fibroblasts support osteoclastogenesis through RANKL, TNF*α*, and IL-7 in an in vitro model derived from human psoriatic arthritis. *Journal of Pathology*.

[6] Dalbeth N., Smith T., Nicolson B. (2008). Enhanced osteoclastogenesis in patients with tophaceous gout: urate crystals promote osteoclast development through interactions with stromal cells. *Arthritis & Rheumatism*.

[7] Lee S.-J., Nam K.-I., Jin H.-M. (2011). Bone destruction by receptor activator of nuclear factor *κ*B ligand-expressing T cells in chronic gouty arthritis. *Arthritis Research & Therapy*.

[8] Palmer D. G., Highton J., Hessian P. A. (1989). Development of the gout tophus. An hypothesis. *American Journal of Clinical Pathology*.

[9] Palmer D. G., Hogg N., Denholm I., Allen C. A., Highton J., Hessian P. A. (1987). Comparison of phenotype expression by mononuclear phagocytes within subcutaneous gouty tophi and rheumatoid nodules. *Rheumatology International*.

[10] Dalbeth N., Pool B., Gamble G. D. (2010). Cellular characterization of the gouty tophus: a quantitative analysis. *Arthritis & Rheumatism*.

[11] Choe J.-Y., Lee G. H., Kim S.-K. (2011). Radiographic bone damage in chronic gout is negatively associated with the inflammatory cytokines soluble interleukin 6 receptor and osteoprotegerin. *The Journal of Rheumatology*.

[12] Choe J.-Y., Jung H.-Y., Park K.-Y., Kim S.-K. (2014). Enhanced p62 expression through impaired proteasomal degradation is involved in caspase-1 activation in monosodium urate crystal-induced interleukin-1*β* expression. *Rheumatology*.

[13] Horowitz M. C., Xi Y., Wilson K., Kacena M. A. (2001). Control of osteoclastogenesis and bone resorption by members of the TNF family of receptors and ligands. *Cytokine and Growth Factor Reviews*.

[14] Ogasawara T., Katagiri M., Yamamoto A. (2004). Osteoclast differentiation by RANKL requires NF-*κ*B-mediated downregulation of cyclin-dependent kinase 6 (Cdk6). *Journal of Bone and Mineral Research*.

[15] Chiou W.-F., Huang Y.-L., Liu Y.-W. (2014). (+)-Vitisin A inhibits osteoclast differentiation by preventing TRAF6 ubiquitination and TRAF6-TAK1 formation to suppress NFATc1 activation. *PLoS ONE*.

[16] Wagner E. F., Eferl R. (2005). Fos/AP-1 proteins in bone and the immune system. *Immunological Reviews*.

[17] Grigoriadis A. E., Wang Z.-Q., Cecchini M. G. (1994). c-Fos: a key regulator of osteoclast-macrophage lineage determination and bone remodeling. *Science*.

[18] David J.-P., Sabapathy K., Hoffman O., Idarraga M. H., Wagner E. F. (2002). JNK1 modulates osteoclastogenesis through both c-Jun phosphorylation-dependent and -independent mechanisms. *Journal of Cell Science*.

[19] Hotokezaka H., Sakai E., Kanaoka K. (2002). U0126 and PD98059, specific inhibitors of MEK, accelerate differentiation of RAW264.7 cells into osteoclast-like cells. *The Journal of Biological Chemistry*.

[20] Arch R. H., Gedrich R. W., Thompson C. B. (1998). Tumor necrosis factor receptor-associated factors (TRAFs)—a family of adapter proteins that regulates life and death. *Genes & Development*.

[21] Lomaga M. A., Yeh W.-C., Sarosi I. (1999). TRAF6 deficiency results in osteopetrosis and defective interleukin-1, CD40, and LPS signaling. *Genes and Development*.

[22] Liu-Bryan R., Scott P., Sydlaske A., Rose D. M., Terkeltaub R. (2005). Innate immunity conferred by Toll-like receptors 2 and 4 and myeloid differentiation factor 88 expression is pivotal to monosodium urate monohydrate crystal-induced inflammation. *Arthritis and Rheumatism*.

[23] Pope R. M., Tschopp J. (2007). The role of interleukin-1 and the inflammasome in gout: implications for therapy. *Arthritis and Rheumatism*.

[24] Martinon F., Pétrilli V., Mayor A., Tardivel A., Tschopp J. (2006). Gout-associated uric acid crystals activate the NALP3 inflammasome. *Nature*.

[25] So A., De Smedt T., Revaz S., Tschopp J. (2007). A pilot study of IL-1 inhibition by anakinra in acute gout. *Arthritis Research & Therapy*.

[26] Terkeltaub R., Sundy J. S., Schumacher H. R. (2009). The interleukin 1 inhibitor rilonacept in treatment of chronic gouty arthritis: results of a placebo-controlled, monosequence crossover, non-randomised, single-blind pilot study. *Annals of the Rheumatic Diseases*.

[27] Jimi E., Nakamura I., Duong L. T. (1999). Interleukin 1 induces multinucleation and bone-resorbing activity of osteoclasts in the absence of osteoblasts/stromal cells. *Experimental Cell Research*.

[28] Kim J. H., Jin H. M., Kim K. (2009). The mechanism of osteoclast differentiation induced by IL-1. *The Journal of Immunology*.

